# Examining the relationships between cortical maturation and white matter myelination throughout early childhood

**DOI:** 10.1016/j.neuroimage.2015.10.038

**Published:** 2015-10-21

**Authors:** Elise C. Croteau-Chonka, Douglas C. Dean, Justin Remer, Holly Dirks, Jonathan O'Muircheartaigh, Sean C.L. Deoni

**Affiliations:** a Advanced Baby Imaging Lab, Brown University School of Engineering, Providence, RI, 02912, USA; b Center for Biomedical Engineering, Brown University, Providence, RI, 02912, USA; c Waisman Laboratory for Brain Imaging and Behavior, University of Wisconsin-Madison, Madison, WI, 53705, USA; d Department of Neuroimaging, King's College London, Institute of Psychiatry, London, United Kingdom; e Department of Pediatric Radiology, Children's Hospital Colorado, Aurora, CO, 80045, USA; f Department of Radiology, University of Colorado Denver, Aurora, CO, 80045, USA

**Keywords:** Infant brain development, Brain MRI, Cortical thickness, Myelination, Cortical development, Myelin water fraction

## Abstract

Cortical development and white matter myelination are hallmark processes of infant and child neurodevelopment, and play a central role in the evolution of cognitive and behavioral functioning. Non-invasive magnetic resonance imaging (MRI) has been used to independently track these microstructural and morphological changes *in vivo*, however few studies have investigated the relationship between them despite their concurrency in the developing brain. Further, because measures of cortical morphology rely on underlying gray–white matter tissue contrast, which itself is a function of white matter myelination, it is unclear if contrast-based measures of cortical development accurately reflect cortical architecture, or if they merely represent adjacent white matter maturation. This may be particularly true in young children, in whom brain structure is rapidly maturing. Here for the first time, we investigate the dynamic relationship between cortical and white matter development across early childhood, from 1 to 6 years. We present measurements of cortical thickness with respect to cortical and adjacent myelin water fraction (MWF) in 33 bilateral cortical regions. Significant results in only 14 of 66 (21%) cortical regions suggest that cortical thickness measures are not heavily driven by changes in adjacent white matter, and that brain imaging studies of cortical and white matter maturation reflect distinct, but complimentary, neurodevelopmental processes.

## Introduction

Two important neurodevelopmental processes that occur throughout infancy and early childhood are the maturation of myelinated white matter and the development of the cerebral cortex. The formation of the lipid bilayer myelin sheath around neuronal axons (myelination) is essential for the rapid brain messaging required for higher order behavioral and cognitive functioning. Brain disconnectivity resulting from aberrant or insufficient development of the myelin sheath may underlie a number of neuropsychiatric disorders, including autism and attention deficit hyperactivity disorder (Belmonte et al., 2004; Krain and Castellanos, 2006; Konrad and Eickhoff, 2010; Xiao et al., 2014). Measures of cortical development, including changes in thickness, surface area, gyrification, volume, and gray matter myelination, have also been investigated in the context of developmental and psychiatric disorders (Courchesne et al., 2007; Hazlett et al., 2012), and in relation to cognitive performance (Shaw et al., 2007, 2012).

Advances in magnetic resonance imaging (MRI) have allowed for the *in vivo* investigation of myelination and cortical maturation both across development and in association with cognitive and behavioral development. Multicomponent relaxometry (MCR) techniques, such as mcDESPOT (multicomponent driven equilibrium single pulse observation of T_1_ and T_2_) (Deoni et al., 2008), enable the visualization and quantification of a surrogate measure of myelin content, termed the myelin water fraction (MWF). MCR decomposes the measured MRI signal into the contributions of signal signatures associated with differing microanatomical water compartments. In the brain, three distinct water pools are commonly observed, corresponding to the free intra and extra-axonal water, the CSF water, and the water trapped between lipid bilayers of the myelin sheath (MacKay et al., 2006). Quantification of the myelin-associated signal, the MWF, is a useful metric for tracking white matter maturation (Deoni et al., 2012; Dean et al., 2015) and its relationship to cognitive development (O'Muircheartaigh et al., 2013, 2014; Deoni et al., 2014) in the developing brain.

Most commonly measured through Freesurfer segmentation (Fischl, 2012) of the cortical ribbon from a T_1_-weighted MR image, cortical thickness is an oft used metric for tracking synaptic density and cortical maturation. While Freesurfer analysis is not formally recommended for use in children under 4 years of age, it has been utilized in investigations of cortical development in infants and toddlers as young as 12 months (Lowe et al., 2012; Travis et al., 2014). Accurate and reproducible delineation of cortical gray matter from underlying and adjacent white matter is a prerequisite for calculating cortical thickness. In ours (Deoni et al., 2015), and others (Lyall et al., 2014) experience, inaccuracies in cortical segmentation can be attributed to insufficient gray–white matter contrast in children under 12 months.

While myelination and cortical development do not occur independently, with both processes occurring symbiotically during the development of neural systems, few studies have sought to investigate the relationship between them. Further, since accurate cortical thickness measures necessitate strong gray–white matter image contrast, which itself is a function of white matter myelination, it is unclear if measures of cortical thickness in early childhood reflect cortical architecture or adjacent white matter maturation. In this work, we aim to directly examine the relationships between cortical thickness and white matter myelination in a large cohort of 134 typically-developing children between 1 and 6 years of age. We measured cortical thickness and calculated the MWF within directly adjacent white matter in 33 bilateral cortical regions. Our results show that cortical thickness changes are not fully explained by MWF changes alone, suggesting that Freesurfer cortical thickness values and MWF are measuring distinct and complementary processes of neurodevelopment.

## Materials and methods

### Study design and participants

**Fig. 1:**
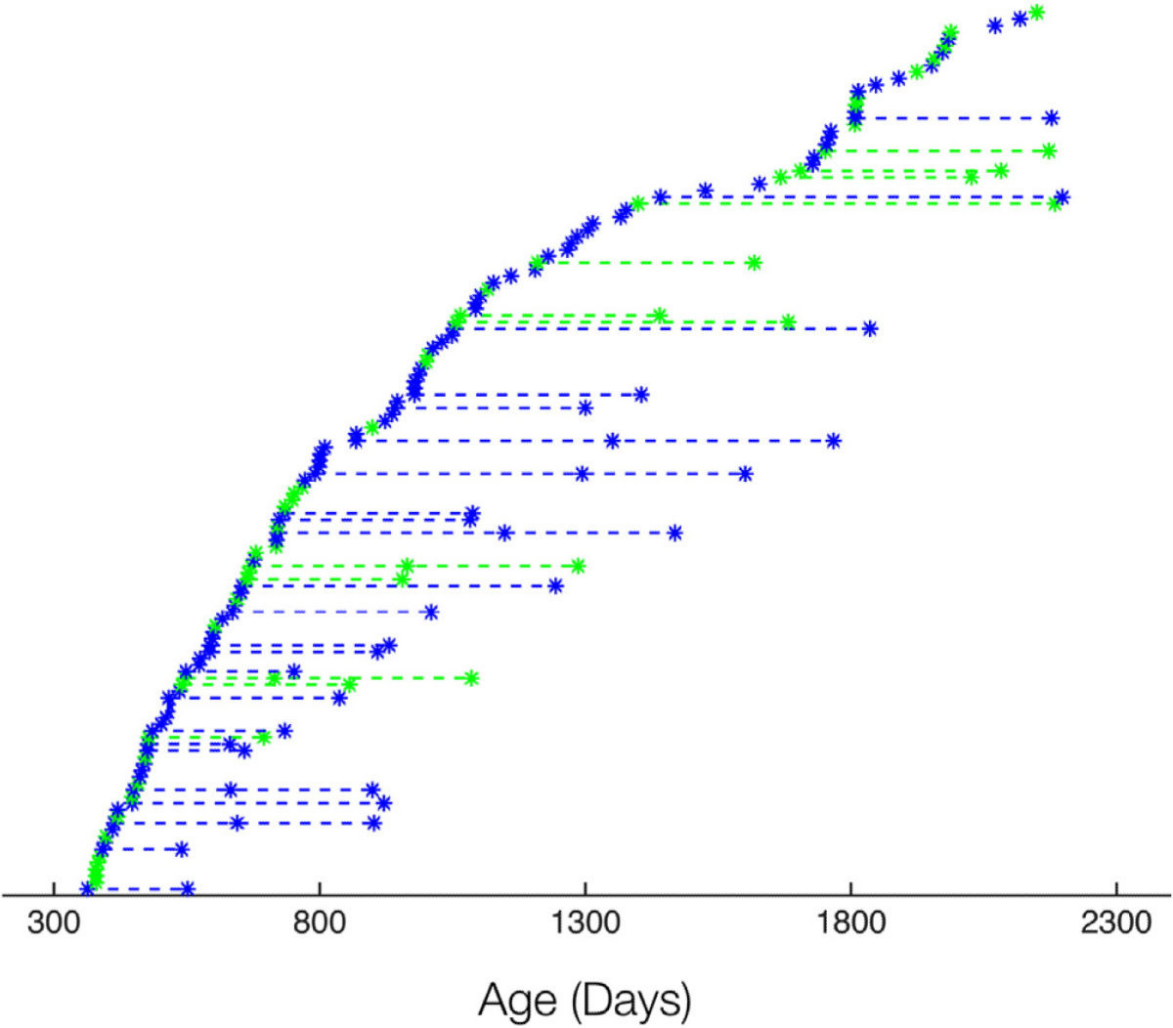
Age distribution (corrected to a 40-week gestation) of study cohort with females in green and males in blue. Individual scans are denoted by an asterisk, with dashed lines connecting repeated measurements from the same child.

Data from 134 (58 female) healthy and typically-developing children approximately 1 to 6 years of age (363 to 2198 days corrected to a 40-week gestation) were used in this study. These children were recruited as part of an ongoing longitudinal investigation of white matter maturation in relation to behavioral development in infancy and early childhood (Deoni et al., 2012). Full demographic information is provided in Table 1. A total of 177 scans were performed, with 36 children scanned at least twice and 7 children scanned three times. The average time between repeat scans was approximately one year (Fig. 1). Inclusion criteria consisted of: birth between 37 and 42 weeks gestation; no abnormalities present on fetal ultrasound; no delivery complications (*i.e.* no visits to the neonatal intensive care unit); APGAR score of 8 or higher; no *in utero* exposure to illicit drugs or alcohol; no pregnancy complications (*i.e.* preeclampsia); no familial history of learning disability, behavioral or psychiatric disorder; and no reported neurological events or disorders in the infant such as head trauma or epilepsy. Child, sibling, and parent medical histories were collected as a supplement to parental interviews conducted at the time of study enrollment. Written informed consent was obtained from the parent(s) or legal guardian of each participating child, and all experimentation was performed under the approval of the Brown University Institutional Review Board.

### Image acquisition

To measure MWF, whole-brain mcDESPOT data were acquired using age-optimized imaging protocols described previously (Deoni et al., 2012) and summarized in Table 2. All imaging was performed on a 3T Siemens Tim Trio scanner with a 12-channel head RF coil array. The data set for each child includes 8 T_1_-weighted spoiled gradient echo (SPGR) images, 2 inversion-prepared SPGR images (IR-SPGR), and 2 sets of T_1_/T_2_-weighted steady-state free precession (bSSFP) images, each acquired with a differing radio-frequency phase-cycling pattern (Deoni, 2011). High resolution volumetric T_1_-weighted MP-RAGE data were also acquired for cortical morphometry analysis.

Children under the age of four were imaged during natural (non-sedated) sleep, while children over four were imaged while watching a favorite movie or TV show (Dean et al., 2014). To attenuate noise levels in the scanner, and keep the youngest participants asleep for the duration of the session, peak gradient amplitudes and slew rates were reduced to 25 mT/m/s, foam inserts (Quiet Barrier HD Composite, UltraBarrier USA) were secured inside the scanner bore, and headphones (MR Confon, Germany) were positioned to cover the ears. To limit the possibility of movement during the scan, all children were swaddled in age-appropriate MedVac vacuum immobilization bags (CFI Medical Solutions, USA) and their heads were kept in place with foam pads. A research assistant watched over infants from inside the scanner room, and additional monitoring was possible using a pediatric pulse-oximetry system and infrared camera. During acquisition, image data was evaluated for motion artifacts including blurring and ghosting. Presentation of these artifacts on an image necessitated repeated acquisition of that image using the original FOV positioning and sequence parameters until higher quality data was obtained. These motion-free images were then incorporated into the child's data set as replacements for artifact-laden images prior to image processing (Dean et al., 2014).

### Image analysis

#### Generation of myelin water fraction maps

Following image acquisition, the mcDESPOT data from each child were linearly co-registered to account for subtle intra-scan motion (Jenkinson et al., 2002), non-brain signal was removed (Smith, 2002), B_0_ and B_1_ field calibration maps were calculated (Deoni, 2011), and voxel-wise T_1_ and MWF maps were calculated using an iterative stochastic region contraction approach (Deoni and Kolind, 2014).

#### Delineation of cortical regions

Low frequency intensity inhomogeneities were removed from the high resolution T_1_-weighted images using Advanced Normalization Tools (ANTs) nonparametric non-uniform normalization (N3) bias correction. Freesurfer (Fischl, 2012) was then used to demarcate the cortex into 33 distinct regions per hemisphere and segment the cortical ribbon for cortical thickness calculations. At each stage in the Freesurfer processing pipeline, the MP-RAGE T_1_-weighted images were visually inspected and, if needed, manually edited and corrected. This included inspecting data for poor skull-stripping, the additional use of gcut (http://freesurfer.net/fswiki/FsTutorial/SkullStripFix_freeview) and, in extreme cases, manual removal of remaining dura, eye, and other non-brain signal. Mean cortical thickness values were obtained for each region.

#### Calculation of adjacent white matter MWF

To obtain measures of the mean adjacent white matter MWF for each cortical region, each region mask was first blurred with a 2 dimensional Gaussian kernel with a 4 mm FWHM. Non-white matter signal was removed by subtracting the cortex and any other non-brain regions. This blurred mask was then superimposed on the aligned MWF map and the mean MWF value was calculated.

#### Calculation of cortical MWF

Mean MWF values were obtained by co-registering each child's MWF map to their high resolution T_1_ weighted image and superimposing each delineated region onto this registered map. Non-linear registration (Avants et al., 2011) was performed first using the high flip angle T_1_ weighted SPGR image acquired as part of the mcDESPOT protocol, with the transformation matrix subsequently applied to the MWF map. Mean and standard deviation MWF values were calculated for each region, in each hemisphere, for each child.

Similar analysis was performed for the quantitative T_1_ maps calculated as part of the mcDESPOT processing, with mean T_1_ values obtained for each of the 66 cortical and adjacent white matter regions.

**Fig. 2:**
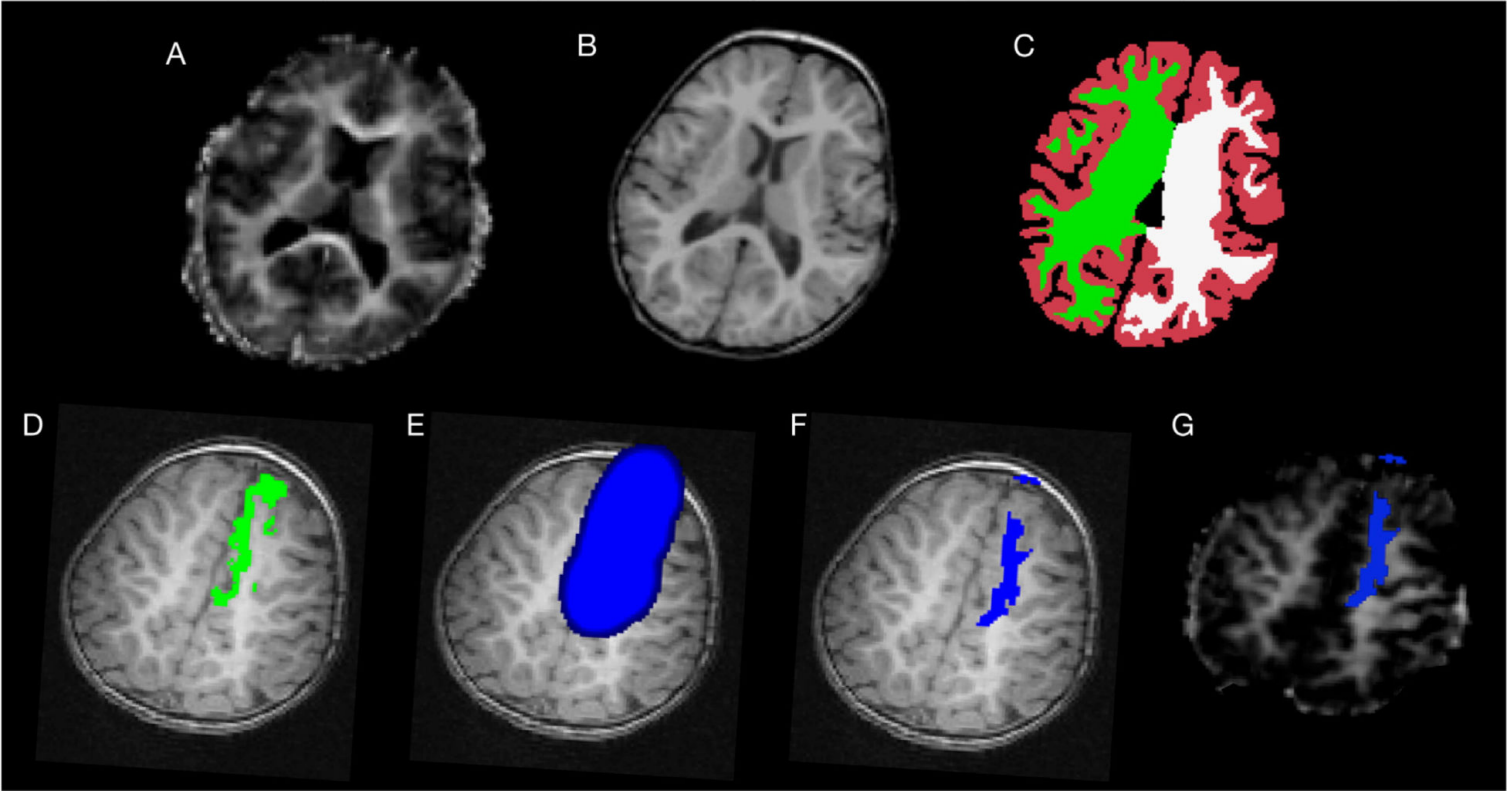
Image analysis steps. (a) MWF maps were calculated using an iterative stochastic region contraction approach. Freesurfer (Fischl, 2012) was used to demarcate the cortex (b) into 33 distinct regions per hemisphere and segment the cortical ribbon (c) for cortical thickness calculations. Freesurfer-derived cortical regions (d) were blurred with a 4 mm FWHM Gaussian kernel (e), and then gray matter and non-brain portions were removed (f). The final mask was then superimposed on to the co-registered MWF image (g) and mean white matter MWF was calculated.

A visual overview of each image analysis step is provided in Fig. 2.

### Region development trajectories

For each region, plots of (1) cortical thickness and adjacent white matter MWF *vs.* age; (2) cortical thickness and cortical MWF *vs.* age; and (3) cortical and adjacent white matter MWF *vs.* age were generated. We fit continuous logarithmic functions to the MWF and cortical thickness data in these plots, verifying the most appropriate fit to the cortical thickness data using the Bayesian Information Criterion (BIC).

To examine the relationships between measures, we first removed the effect of age on each measure by fitting the appropriate trend and subtracting it from the raw values (*i.e.*, calculating the residuals). We then calculated the Pearson product–moment correlation coefficient between these residuals for (1) cortical thickness *vs.* adjacent white matter MWF; (2) cortical thickness *vs.* cortical MWF; and (3) cortical *vs.* adjacent white matter MWF. The first of these analyses examined whether the maturation of adjacent myelin is a significant driver for cortical growth; the second sought to determine if changes in cortical myelin content are a significant driver for cortical growth; and the third explored the strength of the association between cortical and adjacent white matter development. We accounted for multiple comparisons using the Holm–Bonferroni method with an alpha of 0.05 (corrected for 33 bilateral region comparisons).

Using the average longitudinal relaxation (T_1_) times for each of the 66 cortical and adjacent white matter regions, we also calculated the expected ideal (*i.e.*, without contaminating proton density effects) T_1_-weighted signal as (1–2e^–TI/T1^), with TI = 950 ms, matching the TI of the acquired MP-RAGE data. Gray–adjacent white matter T_1_ contrast was then calculated for each of the 66 regions, and this contrast plotted against the region's mean cortical thickness. This analysis was performed to determine the sensitivity of, and relationship between, cortical thickness measures and ideal image contrast. As before, the Pearson's *r* was calculated and assessed for significance using the Holm–Bonferroni method with an alpha of 0.05.

## Results

**Fig. 3:**
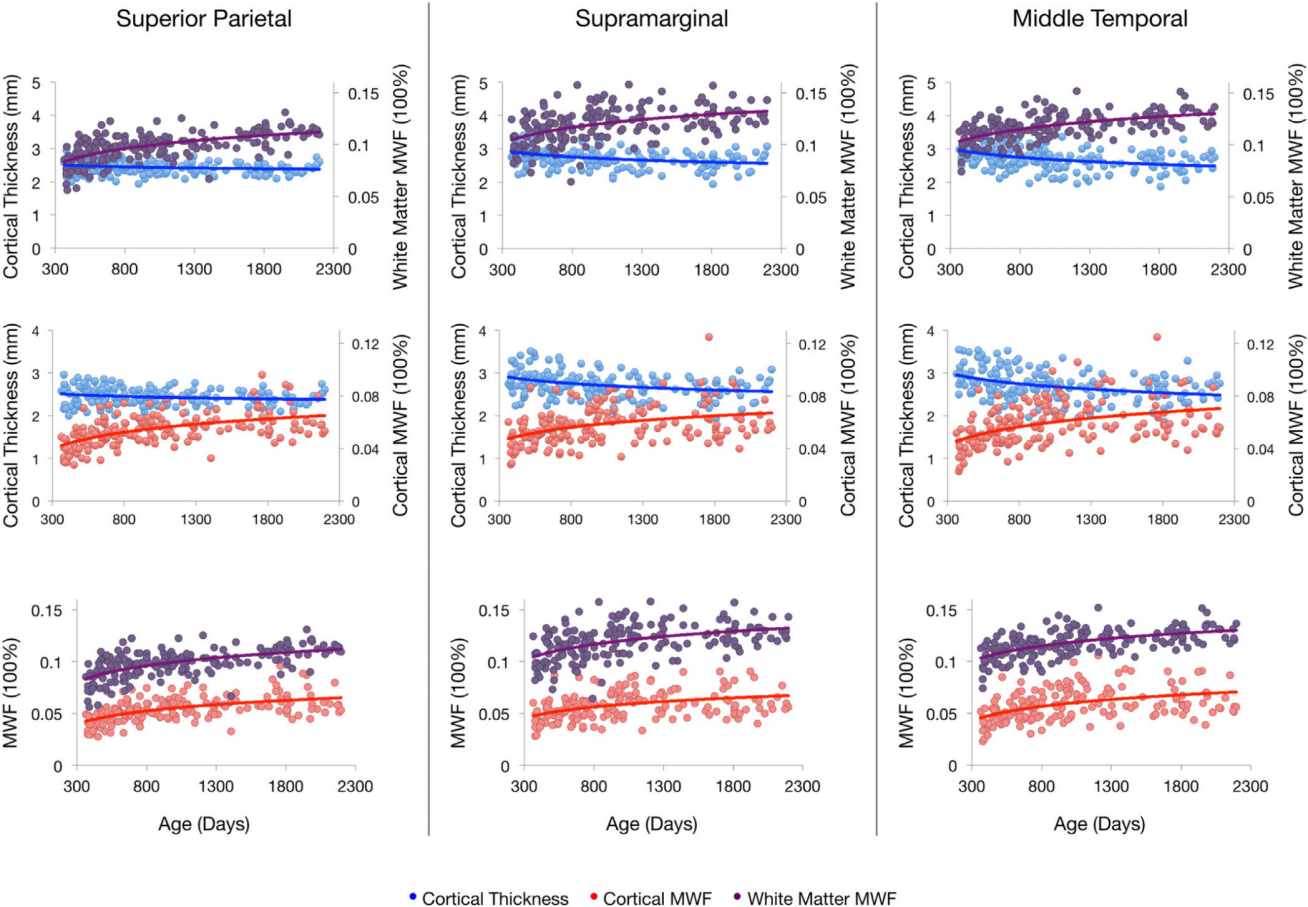
Maturation profile comparisons between cortical thickness, cortical MWF, and adjacent white matter MWF in the left hemisphere of three cortical regions that vary in rate of cortical thinning. Similar trends are observed in the right hemisphere of these regions and in both hemispheres of the remaining 30 bilateral regions not pictured.

Fig. 3 shows raw data plots and superimposed growth models for (1) cortical thickness and adjacent white matter MWF *vs.* age; (2) cortical thickness and cortical MWF *vs.* age; and (3) cortical and adjacent white matter MWF *vs.* age for a representative subset of slow, moderate, and fast developing cortical regions. In agreement with prior data obtained by our group (Deoni et al., 2012, 2015), we model the development of both cortical and adjacent white matter MWF using an increasing logarithmic function. The fit curve equations in Table 3 reveal a range in the logarithmic slope (*i.e.* the rate of MWF development) across cortical regions. Absolute cortical myelin content values are, overall, lower compared to adjacent white matter, as expected.

To investigate how cortical thickness changes with age, we fit logarithmic, quadratic, and linear growth models to the data and compared them using the BIC. While some regions were more appropriately characterized by linear or quadratic fits, the majority of regions follow a logarithmic trajectory (Table 4). To us, these results justified global logarithmic modeling of cortical thickness for all subsequent analyses. Apart from 5 regions (bilateral entorhinal, right parahippocampal, and bilateral temporal pole), measures of cortical thickness follow a decreasing trajectory with age.

**Fig. 4:**
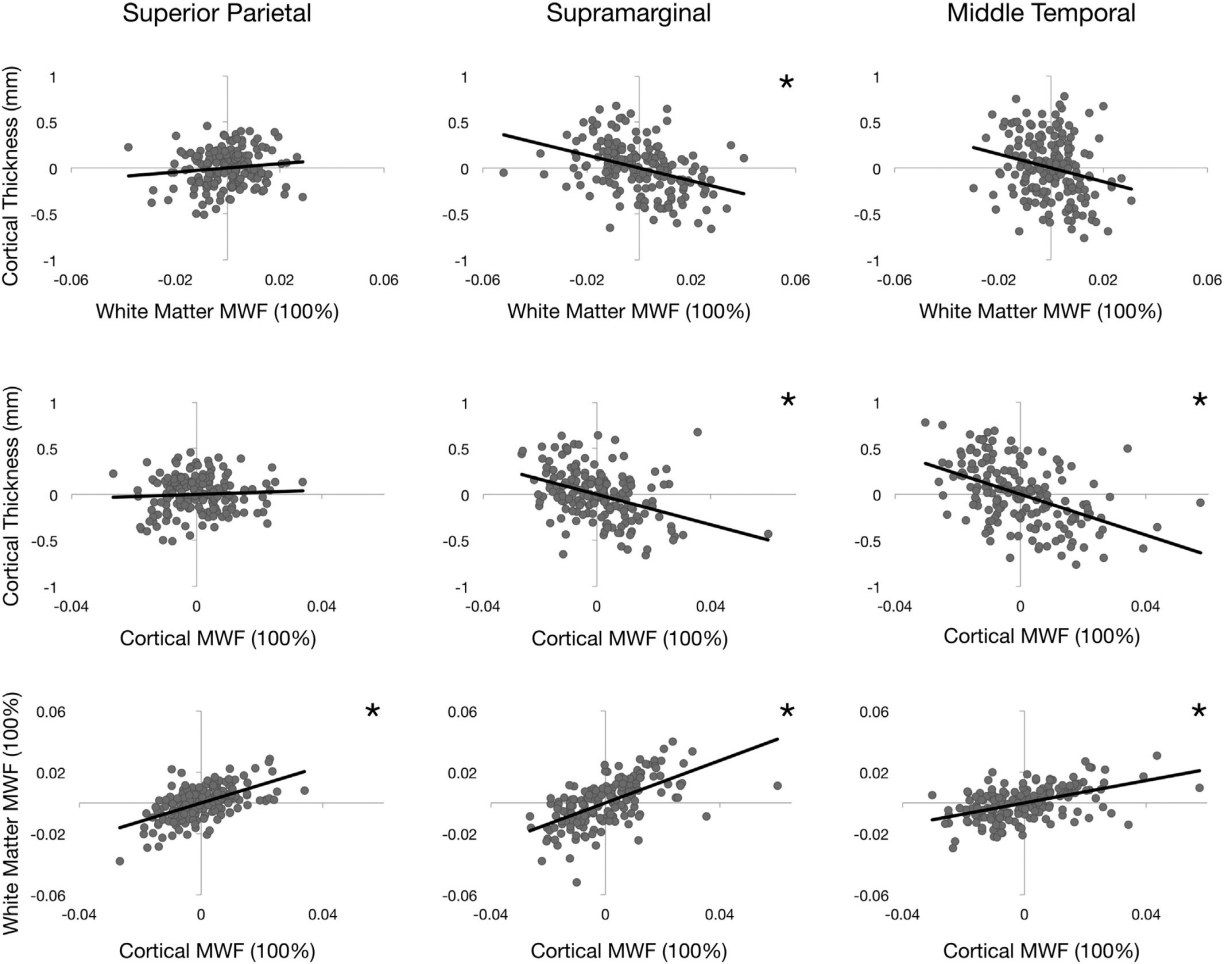
Comparisons between cortical thickness, cortical MWF, and adjacent white matter MWF residual values obtained by subtracting the logarithmic model predictions from measured values shown in Fig. 2. Asterisks denote a statistically significant (p < 0.05 corrected for multiple comparisons) relationship between the two measurements shown in a given plot.

The relationships between cortical thickness, adjacent white matter MWF, and cortical MWF are shown in Fig. 4, which contains plots of the residuals for each measure against the others, and Table 5, which details quantitative results from correlation analyses. By examining the residuals (calculated by subtracting the logarithmic model predictions from measured values), we removed the effect of age from the data. Comparing changes in cortical thickness with those of adjacent white matter reveals a significant (p < 0.05 corrected for multiple comparisons) *negative* relationship between these processes in 10 of 66 regions (Pearson's *r* range: −0.374 to −0.252), including the inferior parietal, supramarginal, rostral middle frontal, and superior frontal regions bilaterally. A significant *positive* relationship was found in 4 of 66 regions (Pearson's *r* range: 0.249 to 0.29), including the right cuneus, right lingual, and bilateral transverse temporal regions.

In 16 of 66 total regions, we found a statistically significant (p < 0.05 corrected for multiple comparisons) *negative* relationship between cortical thickness and cortical MWF (*i.e.* greater thickness is associated with lower MWF). Correlation coefficients for these relationships range from −0.248 to −0.474. Bilateral significance in the pars triangularis, caudal middle frontal, middle temporal, inferior parietal, inferior temporal, and supramarginal regions accounts for 12 of these results. The remaining 4 significant relationships are found in the right postcentral, left rostral middle frontal, left superior temporal, and left parsopercularis regions. Finally, cortical MWF and white matter MWF show significant *positive* relationships (Pearson's *r* range: 0.209 to 0.742) in 63 of 66 regions. Here, the only non-significant regions are the left entorhinal cortex and bilateral temporal pole.

**Fig. 5:**
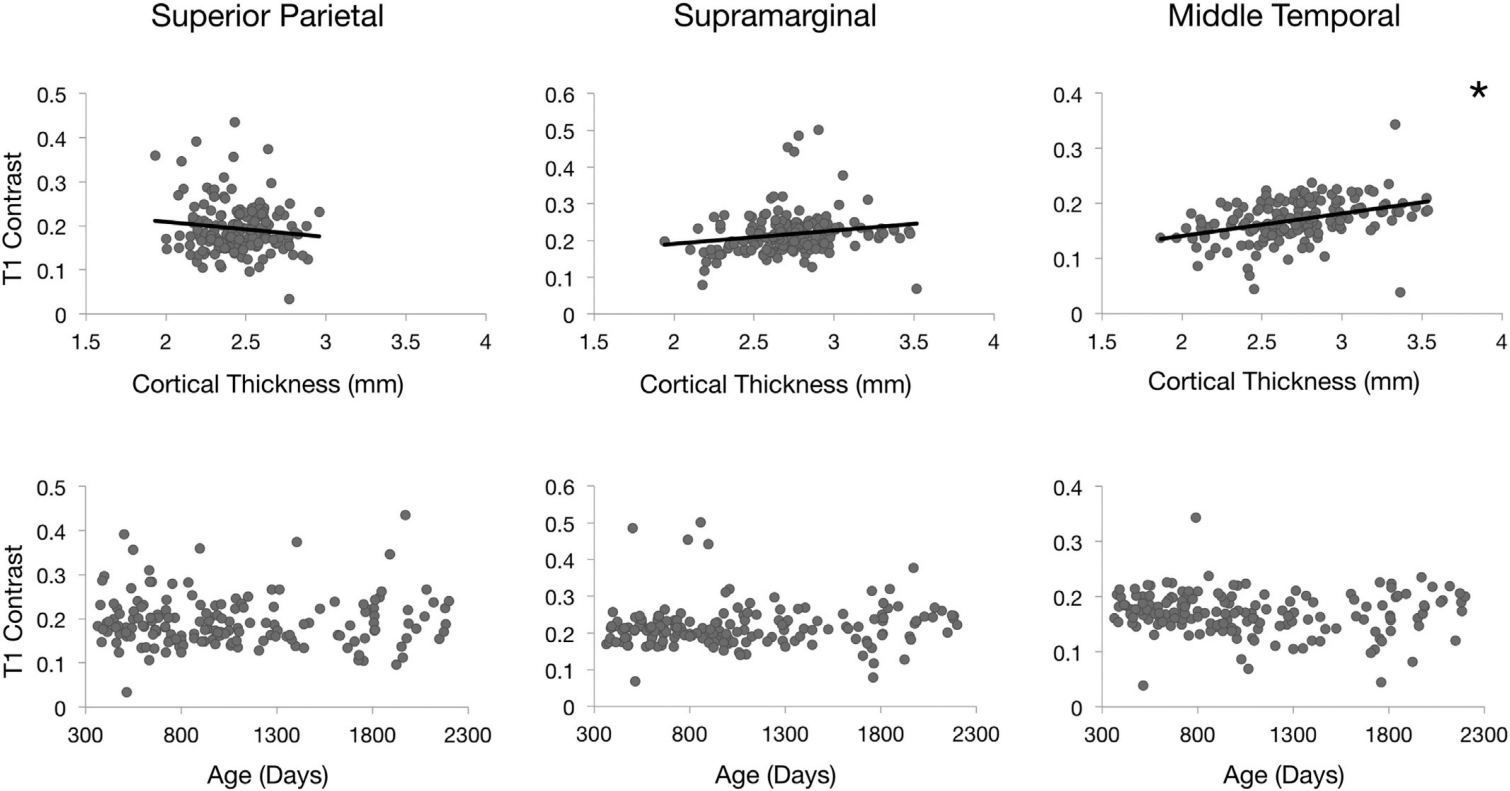
Comparisons between T_1_ contrast and cortical thickness in the left hemisphere of three cortical regions that vary in rate of cortical thinning. The second row shows plots for T_1_ contrast against age in these regions. Denoted by the asterisk, a statistically significant (p < 0.05 corrected for multiple comparisons) relationship exists between T_1_ contrast and cortical thickness in the left middle temporal region.

Significant relationships (p < 0.05 corrected for multiple comparisons) between T_1_ contrast and cortical thickness exist in 10 of 66 regions, including both hemispheres of the inferior parietal, middle temporal, and pars orbitalis regions. Globally, T_1_ contrast varies only subtly between 1 and 6 years of age. Similarly to Figs. 3 and 4, Fig. 5 highlights this analysis for the left hemisphere superior parietal, supramarginal, and middle temporal regions.

## Discussion

In this work, we have investigated the dynamic relationship between cortical development and white matter maturation using quantitative high resolution and MWF imaging for the first time. In a large cohort of 134 (58 female) healthy and typically-developing children, we show that cortical thickness, cortical myelin, and adjacent white matter myelin each follow logarithmic development trajectories. Myelin trajectories presented here are consistent with our prior investigations (Deoni et al., 2012, 2015) and cortical thickness trajectories were chosen through BIC analysis. Visual inspection of residual plots revealed approximately normal distributions of points around the origin, providing additional evidence in favor of these models. In 61 of the 66 regions examined, cortical thickness is found to decrease logarithmically from 1–6 years of age. Prior studies have demonstrated early expansions in cortical development from birth to 1 year of age, and region-specific cortical thinning from 1–2 years of age (Lyall et al., 2014). Our results reveal that visual, motor, and somatosensory areas appear to have faster rates of cortical thinning compared to frontal and association regions, although future studies are needed to verify these trends. These changes in cortical thickness occur simultaneously with logarithmic increases in both cortical MWF and adjacent white matter MWF. Prior work has revealed a similar relationship between cortical thinning and brain growth in children between the ages of 5 and 11 (Sowell et al., 2004). While the authors suggest that this is perhaps due to increased cortical myelination in lower cortical layers, our analysis presents a more complex picture of cortical development and myelination during the first few years of life.

In particular, we find that cortical development is significantly correlated with both cortical white matter and adjacent white matter maturation in relatively few regions. These relationships are not concentrated in one brain area but instead can be found across the brain in regions that differ in rates of cortical thinning. Notably, these regions include later-myelinating frontal and association regions such as the inferior parietal, supramarginal, rostral middle frontal, and caudal middle frontal regions. When considering all brain regions, however, the square of the correlation coefficient, *r^2^*, between cortical thickness and adjacent white matter MWF does not exceed 0.14. In this case, the linear regression model used to illustrate the relationship between the residual measurements only accounts for 14% of the variability in the data, suggesting that cortical thickness and adjacent white matter MWF are not merely proxies for one another. Similarly, at most only 23% of the data is accounted for when explaining cortical thickness changes with respect to cortical MWF maturation. Combining these results with our observation that the majority of cortical regions show non-significant correlations suggests that measures of cortical thickness and MWF are complimentary, but do not characterize identical underlying processes.

Further support for this claim comes from an analysis of the relationship between cortical thickness and gray–white matter T_1_ contrast. Across early childhood (specifically up to 5 years of age), white matter myelination advances in a caudal–cranial, posterior–anterior pattern. In combination with changes in fiber density and coherence, compartmentalization of free water, and changes in macromolecule, protein, lipid, and cholesterol content, this maturation results in significant reductions in white matter relaxation parameters (T_1_ and T_2_). Analogously, changes in synaptic density and cortical architecture also result in widespread reductions in cortical T_1_ and T_2_ (Deoni et al., 2015). Combined, these changing MRI parameters yield a maturing gray–white matter tissue contrast that gradually takes on an adult-level appearance (Barkovich et al., 1988; Paus et al., 2001). Knowing that myelination contributes to changes in T_1_, evidence of a relationship between T_1_ contrast and cortical thickness could be suggestive of a developmental connection between myelination and cortical thickness. However, our analysis shows little evidence of such a relationship. This lends support to measures of cortical thickness being independent of white matter MWF. Looking further at T_1_ contrast values over time, we also see that adult levels of contrast are established and relatively stable by 1 year of age, compared to the increasing logarithmic trajectory of both cortical and white matter MWF from 1 to 6 years of age. This may suggest that myelination is not the primary driver of T_1_ contrast within this age range, a conclusion supported by prior null findings of a MWF–T_1_ relationship in white matter across childhood (Deoni et al., 2012; Harkins et al., 2015).

A potential methodological concern with this work lies in the relatively low resolution of the Freesurfer (Fischl, 2012) segmented cortical regions. To ensure accurate parcellation, images were visually inspected at each stage in the processing pipeline. Children under the age of 1 were also excluded from this work due to insufficient gray–white matter contrast observed in this age range. While lower than the recommended 1 mm^3^ isotropic resolution for adult studies, the (1.2 × 1.2 × 1.2) mm^3^ spatial resolution of our T_1_w images either meets or exceeds resolutions used in prior pediatric neuroimaging studies (Deoni et al., 2015; Shaw et al., 2012).

Multicomponent relaxometry techniques, such as mcDESPOT, are specific to early myelin development (Deoni et al., 2008, 2012, 2013). White matter microstructural changes, however, extend beyond myelination and encompass changes in axon number and density. Diffusion tensor imaging (DTI) can provide insight into these additional neuroanatomical measures, but sacrifices myelination specificity (Mädler et al., 2008; Yoshida et al., 2013). Future studies using diffusion tensor imaging (DTI) in combination with mcDESPOT are needed to gain a more comprehensive understanding of early white matter development in this age range.

While this work highlights primarily non-significant relationships between measures of cortical development and white matter maturation, a temporal offset may exist between these processes that was not considered here. Prior work has shown that over time, trajectories of cortical thickness changes are regionally-dependent and are associated with cognitive development and outcome (Shaw et al., 2006). Further analysis of both morphological and behavioral measures is necessary to examine whether early changes in cortical thickness may predict later changes in MWF, or *vice versa.* Gender is another factor that was not considered here. We have previously shown (Deoni et al., 2015) no significant evidence for sexual dimorphism in cortical MWF and T_1_ development trajectories or mean values from 1 to 6 years of age. While sex-specific differences in the magnitude of cortical thickness have been observed from age 6 into adulthood, rate of cortical thickness change does not show gender influences in this period (Raznahan et al., 2011). Gender differences in cortical development and white matter maturation relationships may, therefore, be best investigated in late childhood and early adolescence, which is beyond the scope of this work.

## Conclusions

Our results show that changes in cortical thickness from 1–6 years of age are non-linear and largely independent of both cortical and adjacent white matter maturation. These findings raise questions about the degree to which other cortical measures explain the relationship between cortical and white matter development. While further investigation is needed to determine if the regional variation in cortical thickness shown here can be linked to cognitive and behavioral outcomes, our results fill in the knowledge gap on cortical and white matter development trajectories and their relationship to one another in early childhood.

## Figures and Tables

**Table 1:** Participant demographic information.

Gender	Male (n)	76
	Female (n)	58
Racial background	Caucasian (n)	89
	African American (n)	11
	Asian (n)	2
	Mixed Race (n)	18
	Unknown (n)	16
Ethnic background	Hispanic (n)	28
	Non-Hispanic (n)	10
	Unknown (n)	96
Mean age (days)	1044 ± 523	
Age range (days)	363-2198	
Mean gestation (weeks)	39 ± 1.4	
Mean birth weight (lbs)	6.9 ± 1.0	
Mean maternal SES	5.9 ± 1.1	

**Table 2:** Age-optimized imaging protocols.

		12–16 months	16–24 months	24–36 months	36–60 months
SPGR	Field of View (cm)	17 × 17 × 14.4	18 × 18 × 15	20 × 20 × 15	20 × 20 × 15
	Voxel Size (mm)	1.9 × 1.9 × 1.8	1.8 × 1.8 × 1.8	1.8 × 1.7 × 1.8	1.7 × 1.7 × 1.8
	TE/TR (ms)	5.7 ms/14 ms	5.2 ms/13 ms	4.8 ms/12 ms	4.5 ms/11 ms
	Flip Angles (degrees)	2,3,4,5,6,7,10,14	2,3,4,5,6,7,10,14	2,3,4,5,6,8,12,16	3,4,5,6,7,9,13,18
	Bandwidth (Hz/pixel)	350	350	350	350
	Image matrix	96 × 96 × 80	104 × 104 × 84	112 × 112 × 84	112 × 112 × 84
IR-SPGR	TI/TE/TR (ms)	(600, 900) ms/5.7 ms/14 ms	(550, 850) ms/5.2 ms/13 ms	(500, 850) ms/4.8 ms/12 ms	(450, 750) ms/4.5 ms/11 ms
	Flip angle (degrees)	5	5	5	5
	Image matrix	96 × 96 × 40	108 × 104 × 42	112 × 112 × 42	112 × 112 × 42
bSSFP	TE/TR (ms)	5.55 ms/11.1 ms	5.258 ms/10.52 ms	5 ms/10 ms	4.585 ms/9.17 ms
	Flip angles (degrees)	12, 16, 19, 23, 27, 35, 50, 70	12, 16, 19, 23, 27, 35, 50, 70	12, 16, 19, 23, 27, 35, 50, 70	12, 16, 19, 23, 27, 35, 50, 70
	Bandwidth (Hz/pixel)	350	350	350	351
	Image matrix	96 × 96 × 80	104 × 104 × 84	112 × 112 × 84	112 × 112 × 84
High resolution IR-SPGR	Field of view (cm)	17 × 17 × 14.4	18 × 18 × 15	20 × 20 × 15	20 × 20 × 15
	TI/TE/TR (ms)	950 ms/6.9 ms/16 ms	950 ms/6.9 ms/16 ms	950 ms/6.9 ms/16 ms	950 ms/6.9 ms/16 ms
	Flip angle (degrees)	5	5	5	5
	Image matrix	144 × 144 × 116	144 × 144 × 124	160 × 160 × 124	160 × 160 × 124

**Table 3:** Coefficients in fit curve equations for left hemisphere cortical brain regions.

Cortical brain region	Cortical thickness		Adjacent white matter MWF		Cortical MWF	
	Logarithmic	Intercept	Logarithmic	Intercept	Logarithmic	Intercept
Caudal anterior cingulate	–0.179	4.566	0.029	–0.07	0.016	–0.058
Caudal middle frontal	–0.23	4.452	0.022	–0.035	0.017	–0.056
Cuneus	–0.267	4.096	0.014	0.005	0.013	–0.031
Entorhinal	0	2.86	0.01	0.025	0.015	–0.057
Frontal pole	–0.09	3.963	0.019	–0.051	0.016	–0.057
Fusiform	–0.22	4.399	0.014	0.011	0.012	–0.024
Inferior parietal	–0.142	3.609	0.011	0.029	0.012	–0.021
Inferior temporal	–0.303	4.902	0.014	0.02	0.019	–0.063
Insula	–0.145	4.5	0.011	0.031	0.014	–0.051
Isthmus cingulate	–0.297	5.172	0.016	0.019	0.009	–0.011
Lateral occipital	–0.196	3.687	0.01	0.034	0.012	–0.008
Lateral orbitofrontal	–0.092	3.908	0.029	–0.087	0.02	–0.086
Lingual	–0.275	4.286	0.014	0.01	0.008	0.003
Medial orbitofrontal	–0.331	5.443	0.024	–0.064	0.02	–0.091
Middle temporal	–0.264	4.514	0.015	0.016	0.014	–0.036
Paracentral	–0.141	3.605	0.017	–0.001	0.01	–0.019
Parahippocampal	–0.061	3.09	0.016	–0.025	0.01	–0.022
Pars opercularis	–0.23	4.488	0.024	–0.037	0.015	–0.049
Pars orbitalis	–0.38	5.952	0.015	–0.001	0.016	–0.06
Pars triangularis	–0.276	4.662	0.026	–0.06	0.016	–0.053
Pericalcarine	–0.199	3.303	0.016	–0.001	0.01	0.001
Postcentral	–0.145	3.26	0.013	0.023	0.011	–0.011
Posterior cingulate	–0.098	3.807	0.023	–0.035	0.013	–0.036
Precentral	–0.099	3.188	0.018	–0.004	0.013	–0.027
Precuneus	–0.137	3.748	0.021	–0.027	0.012	–0.034
Rostral anterior cingulate	–0.494	7.172	0.023	–0.029	0.015	–0.055
Rostral middle frontal	–0.368	5.527	0.03	–0.098	0.019	–0.07
Superior frontal	–0.259	5.21	0.028	–0.083	0.015	–0.056
superior Parietal	–0.071	2.921	0.016	–0.009	0.013	–0.032
Superior temporal	–0.035	2.961	0.017	–0.002	0.012	–0.029
Supramarginal	–0.19	4.026	0.015	0.014	0.011	–0.017
Temporal pole	0.059	2.846	0.004	0.065	0.014	–0.055
Transverse temporal	–0.108	3.326	0.018	–0.011	0.011	–0.011

**Table 4:** Bayesian Information Criterion analysis of different functions describing left hemisphere changes in cortical thickness with age. Bolded values denote the model that best describes the development trajectories.

Cortical brain region	Logarithmic	Quadratic	Linear
Caudal anterior cingulate	**164.18**	168.59	**163.46**
Caudal middle frontal	**98.71**	103.54	100.95
Cuneus	**45.12**	49.44	54.83
Entorhinal	**227.61**	230.47	**227.45**
Frontal pole	**378.37**	383	**378.11**
Fusiform	**0.59**	6.02	7.93
Inferior parietal	**51.05**	52.96	52.85
Inferior temporal	**81.56**	84.78	86.97
Insula	**–29.7**	–25.65	**–30.7**
Isthmus cingulate	**82.17**	88.65	85.32
Lateral occipital	**–30.93**	**–36.74**	–20.85
Lateral Orbitofrontal	**65.43**	66.58	**63.72**
Lingual	**–6.86**	–4.95	3.41
Medial Orbitofrontal	**123.13**	130.4	127.57
Middle temporal	**128.23**	**127.51**	134.35
Paracentral	**51.95**	56.31	**51.19**
Parahippocampal	**208.39**	213.66	208.55
Pars opercularis	**80.85**	86.17	83.89
Pars orbitalis	**224.75**	229.88	225.04
Pars triangularis	**146.71**	152.04	148.06
Pericalcarine	**19.2**	**18.65**	26.44
Postcentral	**–9.1**	–5.43	–7.11
Posterior cingulate	**26.44**	28.68	**24.48**
Precentral	**–64.76**	–60.05	–64.5
Precuneus	**–28.32**	–26.58	**–31.35**
Rostral anterior cingulate	**81.79**	85.94	82.96
Rostral middle frontal	**106.37**	110.89	107.2
Superior frontal	**58.92**	61.61	**56.44**
Superior parietal	**–57.97**	–53.28	–57.94
Superior temporal	**32.07**	36.93	**32**
Supramarginal	**59.84**	62	62.73
Temporal pole	**305.64**	310.5	**305.48**
Transverse Temporal	**138.96**	143.86	**138.72**

**Table 5:** Pearson product-moment correlation analysis between cortical thickness, white matter myelin water fraction, and cortical myelin water fraction. Bolded values denote significant relationships between measures within a given cortical region after performing a Holm-Bonferroni correction for multiple comparisons.

Cortical brain region	Cortical thickness & adjacent white matter MWF		Cortical thickness & cortical MWF		Cortical & adjacent white matter MWF	
	Pearson's r	p value	Pearson's r	p value	Pearson's r	p value
Caudal anterior cingulate	–0.184	0.0144	–0.106	0.162	0.282	**0.000139**
Caudal middle frontal	–0.274	**0.00023**	–0.27	**0.000274**	0.556	**8.88E–16**
Cuneus	0.159	0.0347	0.102	0.177	0.495	**2.41E–12**
Entorhinal	–0.024	0.747	0.007	0.926	0.16	0.0335
Frontal pole	0.202	0.00692	–0.055	0.469	0.51	**4.13E–13**
Fusiform	–0.19	0.0113	–0.146	0.0527	0.44	**8.67E–10**
Inferior parietal	–0.333	**6.11E–06**	–0.32	**1.40E–05**	0.547	**3.33E–15**
Inferior temporal	–0.184	0.0141	–0.416	**8.41E–09**	0.369	**4.24E–07**
Insula	0.05	0.512	–0.063	0.402	0.61	**0**
Isthmus cingulate	0	0.998	–0.007	0.924	0.313	**2.22E–05**
Lateral occipital	–0.139	0.0645	–0.208	0.00552	0.414	**1.02E–08**
Lateral orbitofrontal	0.028	0.709	–0.049	0.515	0.419	**6.19E–09**
Lingual	0.061	0.419	0.131	0.081	0.245	**0.00103**
Medial orbitofrontal	0.172	0.022	0.136	0.0721	0.465	**7.33E–11**
Middle Temporal	–0.239	0.00133	–0.474	**2.70E–11**	0.489	**5.14E–12**
Paracentral	0.097	0.199	–0.038	0.615	0.458	**1.44E–10**
Parahippocampal	0.154	0.0405	0.049	0.52	0.439	**1.01E–09**
Pars opercularis	–0.252	**0.000717**	–0.316	**1.81E–05**	0.518	**1.50E–13**
Pars orbitalis	–0.21	0.00496	–0.237	0.00151	0.382	**1.49E–07**
Pars triangularis	–0.237	0.0015	–0.264	**0.000385**	0.572	**0**
Pericalcarine	0.154	0.0402	0.142	0.0601	0.74	**0**
Postcentral	0.068	0.372	–0.194	0.0098	0.545	**4.00E–15**
Posterior cingulate	–0.076	0.312	–0.125	0.0983	0.312	**2.42E–05**
Precentral	–0.024	0.748	–0.13	0.0843	0.53	**3.20E–14**
Precuneus	0.217	0.00371	0.005	0.943	0.297	**6.11E–05**
Rostral anterior cingulate	–0.209	0.00528	–0.195	0.00912	0.317	**1.73E–05**
Rostral middle frontal	–0.318	**1.66E–05**	–0.257	**0.000562**	0.592	**0**
Superior frontal	–0.28	**0.000164**	–0.144	0.0566	0.553	**1.33E–15**
Superior parietal	0.123	0.102	0.058	0.439	0.566	**2.22E–16**
Superior temporal	–0.239	0.00138	–0.316	**1.83E–05**	0.549	**2.44E–15**
Supramarginal	–0.374	**3.02E–07**	–0.387	**1.01E–07**	0.607	**0**
Temporal pole	0.102	0.178	0.094	0.214	0.177	0.0185
Transverse temporal	0.249	**0.000851**	–0.08	0.287	0.603	**0**
